# Oxytocin receptor is regulated by *Peg3*

**DOI:** 10.1371/journal.pone.0202476

**Published:** 2018-08-14

**Authors:** Wesley D. Frey, Kaustubh Sharma, Terri L. Cain, Katsuhiko Nishimori, Ryoichi Teruyama, Joomyeong Kim

**Affiliations:** 1 Department of Biological Sciences, Louisiana State University, Baton Rouge, LA, United States of America; 2 Department of Molecular and Cell Biology, Graduate School of Agricultural Science, Tohoku University, Miyagi, Japan; University of Bonn, Institute of Experimental Hematology and Transfusion Medicine, GERMANY

## Abstract

Mouse *Peg3* encodes a DNA-binding protein involved in the milk letdown process. In the current study, we tested whether PEG3 controls the expression of the oxytocin receptor gene. According to the results, PEG3 directly binds to a genomic region within the 3rd exon of *Oxtr*, which contains a DNA-binding motif for PEG3. In nursing female mice, removal of PEG3 resulted in the increased expression of *Oxtr* in mammary epithelial cells and also in the hypothalamus. This suggests a repressor role of PEG3 in the expression of *Oxtr* in these tissues. Overall, this study suggests that *Peg3* may function as a direct transcriptional regulator for *Oxtr* expression that acts to moderate the milk letdown process.

## Introduction

*Peg3* (Paternally Expressed Gene 3) is an imprinted gene localized in proximal mouse chromosome 7/human chromosome 19q13.4 [1–3]. This gene encodes a DNA-binding protein with 12 C2H2 zinc finger motifs known to bind to a large number of genomic targets [4–7]. The list of known downstream genes includes *Pgm2l1*, *H19*, *Msl1* and *Msl3*. PEG3 also contains a KRAB-A domain, thus is predicted to be a transcriptional repressor through its interaction with KAP1 [8]. This predicted repression function has been further confirmed through the observation that the expression levels of its downstream genes are usually up-regulated in the tissues of the mouse mutant models targeting *Peg3* [6, 7]. According to the results from mouse knockout (KO) models, *Peg3* is also involved in controlling fetal growth rates and maternal-caring behaviors [9–13]. However, the detailed mechanism by which *Peg3* is involved in these biological pathways is not well understood.

In murine Peg3-KO studies, both nursing females and pups tend to have a problem in milk provision, subsequently causing reduced growth rates in the pups lacking *Peg3* [9–13]. In placental mammals, milk letdown is mediated through oxytocin circuitry involving the peptide hormone oxytocin (*Oxt*) and its receptor, oxytocin receptor (*Oxtr*) [14, 15]. Oxytocin is produced in the supraoptic nucleus (SON) and paraventricular nucleus (PVN) of the hypothalamus. Axonal projections of these nuclei lead to the posterior pituitary gland, where oxytocin is released into the bloodstream, and exerts its effects through binding to oxytocin receptors present in the target tissues, including uterus, ovary and mammary gland [14, 15]. In a prior mutant model of *Peg3*, reduced numbers of Oxt-expressing neurons were initially observed, which was then thought to be a main cause for the milk provision problem [9]. However, this has been later argued by two independent studies reporting that the mutations did not cause any change in the numbers of Oxt-immunoreactive neurons in the hypothalamus [13, 16, 17]. Nevertheless, subsequent experiments confirmed again the presence of the milk provision problem in several Peg3-KO models, and further substantiated that this defect was more pronounced through the conditional mutation of *Peg3* in the mammary gland than in the hypothalamus [12]. This further suggests that *Peg3* might play more significant roles in the mammary gland than in the hypothalamus for the milk provision process [12]. Consistent with this, *Peg3* is expressed not only in the hypothalamus but also in the other tissues, including placenta, uterus, testis, ovary, and mammary gland [1–3].

*Oxtr* is also known to be widely expressed in various tissues, including the ovary, uterus, brain and mammary gland, whereas *Oxt* is mainly expressed in the hypothalamus. Yet, conditional KO of *Peg3* in the mammary gland exhibited similar defects as those observed from the earlier Peg3-KO models, suggesting that *Peg3* may be involved in the oxytocin circuitry not only through *Oxt* in the hypothalamus but also through *Oxtr* in the mammary gland. This possibility was tested in the current study by performing a series of experiments. The results suggest that PEG3 may function as a transcriptional repressor for the expression of *Oxtr*.

## Results

### PEG3 binding to the mouse *Oxtr* locus

**Fig 1:**
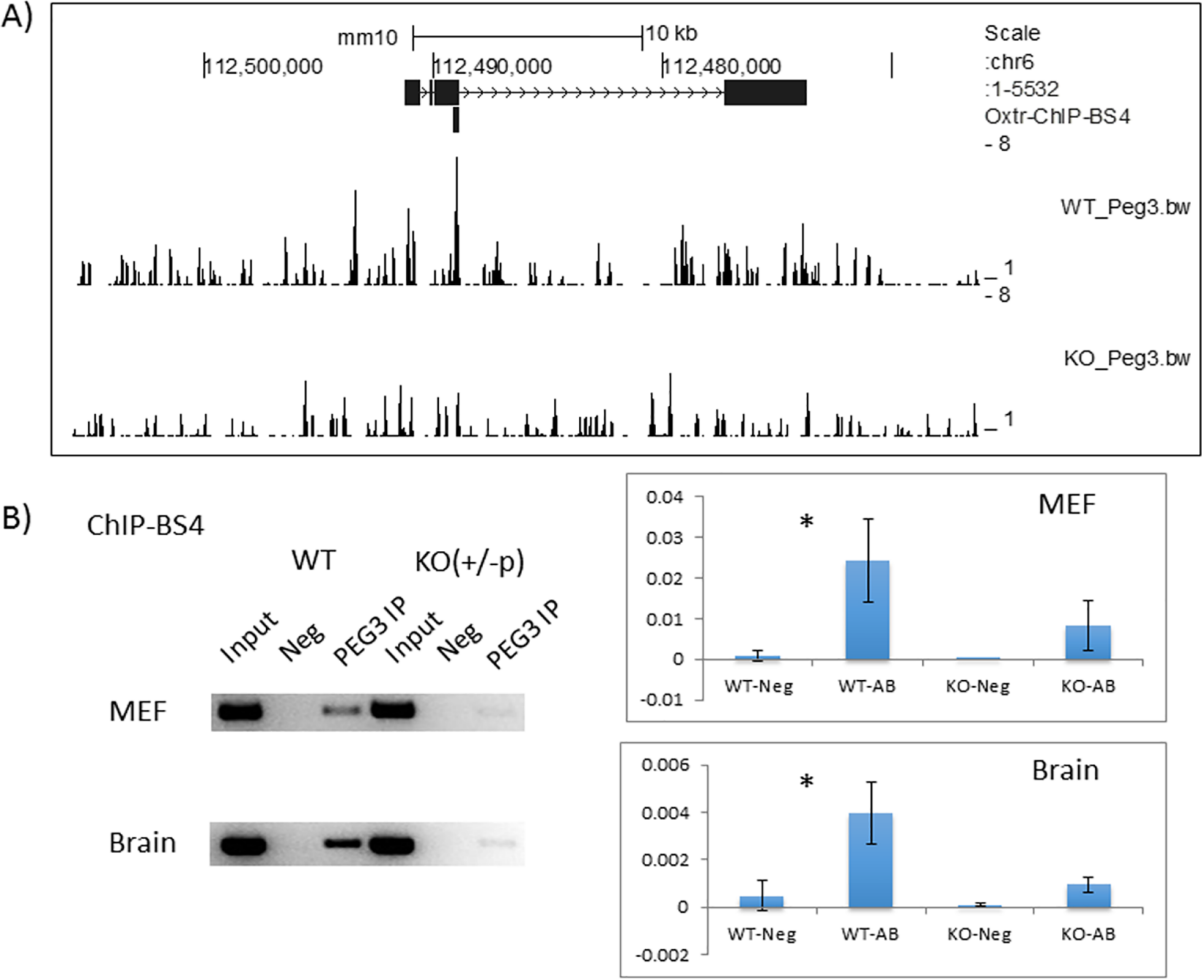
PEG3 binding to the mouse *Oxtr* locus. (**A**) The 40-kb genomic region surrounding the *Oxtr* locus is shown using the UCSC genome browser. The black boxes indicate the 4 exons of *Oxtr*, while the arrows indicate the transcriptional direction of *Oxtr*. The identified potential target region of PEG3 is also indicated with a vertical thick line (Oxtr-ChIP-BS4). Processed ChIP-seq results from WT and KO MEF cells (WT_Peg3.bw and KO_Peg3.bw) were uploaded onto the genome browser to show the enrichment levels by PEG3-ChIP. (**B**) Individual ChIP experiments. The chromatin from MEF and adult brains were immunoprecipitated with anti-PEG3 antibody. The subsequent DNA set, including Input, Negative without any antibody (Neg), the immunoprecipitated DNA with anti-PEG3 antibody (PEG3 IP), were used as templates for a fixed number of PCR cycle to test the *in vivo* binding of PEG3 to the target region (ChIP-BS4). The relative enrichment levels against Input were measured with qPCR experiments and further compared between Neg and PEG3 IP. Asterisks represent statistical significance of the observed differences between Neg and PEG3 IP (*, *p* value < 0.05).

The predicted protein function of PEG3 has been previously tested through performing several ChIP-seq (Chromatin ImmunoPrecipitation-sequencing) experiments [4–6]. For these experiments, we time-mated C57BL/6J females with *Peg3*^*CoKO/+*^ males, and the subsequent 14.5-dpc (day postcoitum) embryos were used for preparing a set of mouse embryonic fibroblast (MEF) cells, *Peg3*^*+/+*^ (WT) and *Peg3*^*+/CoKO*^ (KO) [6]. The chromatin prepared from this set of MEFs were individually immunoprecipitated with anti-PEG3 antibody, and subsequently analyzed with Next Generation Sequencing. This survey identified an initial set of 16 downstream genes, which have been recently published [6]. We further inspected the output by manually scanning the entire set of chromosomes for potential downstream genes. This series of manual inspections identified an additional set of potential genomic targets, including the *Oxtr* locus (**Fig 1A**). At the *Oxtr* locus, a ChIP-seq peak was found within the 3rd exon, which spans a 225-bp genomic region (chr6:112,488,870–112,489,904 in mm10). It is also relevant to note that we were unable to find any significant ChIP-seq peak around the *Oxt* locus (**S1 File**).

Potential binding of PEG3 to the identified region of the *Oxtr* locus was further tested through performing independent ChIP experiments (**Fig 1B**). Several sets of the chromatin from MEFs and brains of WT and KO were immunoprecipitated again with the antibody against PEG3. As shown in **Fig 1B**, the levels of enrichment by the antibody were higher in WT than those from KO-MEF cells, confirming the binding of PEG3 to the target region of the *Oxtr* locus. This was also the case for the brain, which displayed higher levels of enrichment in the WT than in the KO samples. Thus, this series of ChIP experiments confirmed that the identified region is indeed bound by PEG3.

**Fig 2:**
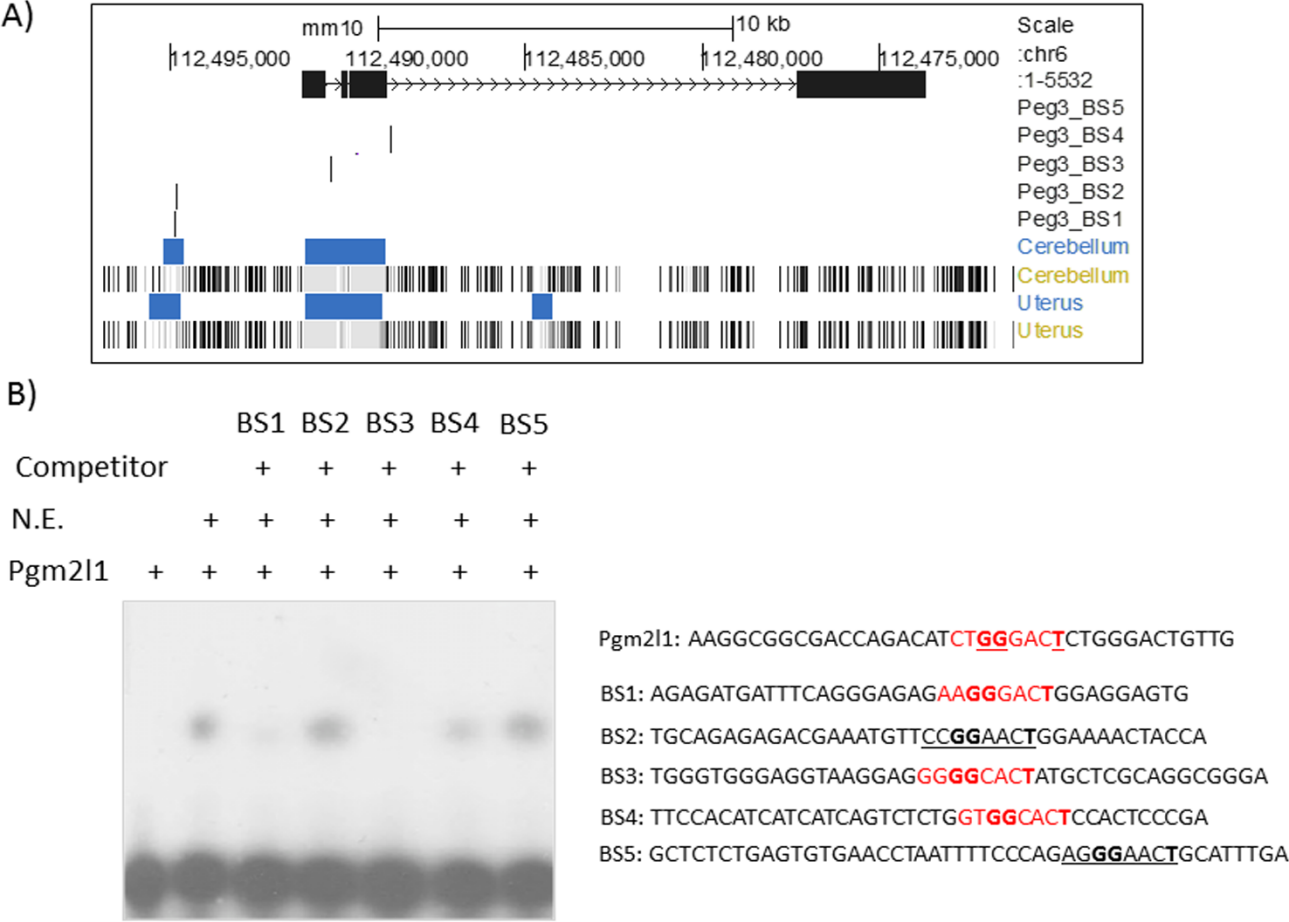
Potential DNA-binding sites of PEG3 within the *Oxtr* locus. (**A**) The 20-kb genomic region of *Oxtr* was scanned with the consensus DNA-binding motif of PEG3, 5'-N-N-G-G-[C/G]-N-[C/G]-T with N being any nucleotide bases, identifying 5 potential binding sites, BS1-BS5. These binding sites are localized within three genomic regions with DNA hypomethylation, which are indicated with blue bars. (**B**) Competitive EMSA (Electrophoretic Mobility Shift Assay). The P^32^-radiolabeled oligonucleotide duplex probe derived from the *Pgm2l1* locus was mixed with mouse brain nuclear extracts (lane 2), and further competed against a set of 5 potential binding sites (lane 3–7). The relative ratio of the labeled Pgm2l1 probe to BS1—BS5 oligonucleotide duplexes was 1 to 200. The sequences of the oligonucleotides used for this assay are shown on right. The potential binding sites are indicated with either underlines or red fonts. The sites with red font indicate the confirmed DNA-binding sites through EMSA, whereas the sites with underlines indicate the predicted but unconfirmed sites. The bold-typed nucleotide bases within each site represent the critical bases for PEG3 binding.

According to previous studies, PEG3 is known to bind to genomic regions with the following consensus DNA motif: 5'-N-N-G-G-[C/G]-N-[C/G-]-T-3' with N indicating any base [5]. Thus, we scanned the 20-kb genomic region of the *Oxtr* locus to identify potential genomic regions with this motif. This manual scanning also focused on the genomic regions with potential regulatory function based on their histone modification and DNA methylation profiles. *Oxtr* is known to be expressed in cerebellum and uterus, thus we selected three genomic regions that are protected from DNA methylation in these tissues. From these regions indicated with blue bars in **Fig 2A**, we have identified 5 potential binding sites, BS1 through 5. PEG3 binding to these potential binding sites was subsequently tested through a series of EMSAs (Electrophoretic Mobility Shift Assays) (**Fig 2B**). For this series of analyses, we used a previously characterized binding site from the *Pgm2l1* locus as a probe [4, 5], and nuclear extracts prepared from mouse brains. Binding of PEG3 to the P^32^-labeled Pgm2l1 oligonucleotide duplex was competed against each candidate DNA-binding site. The results indicated that two candidate sites, BS2 and BS5, did not compete at all, suggesting that these two regions may not contain a DNA-binding site for PEG3. In contrast, the three remaining binding sites, BS1, BS3, and BS4, competed well against the Pgm2l1 probe, suggesting that these regions may have high-affinity DNA-binding sites for PEG3. In particular, BS4 is located within the target region that had already been identified through independent ChIP-seq experiments (**Fig 1**). Thus, this series of analyses confirmed that the *Oxtr* locus has several potential binding sites for PEG3, and that one of these binding sites is indeed bound by PEG3 *in vivo*.

### Mutational effects of *Peg3* and *Oxtr* on animal survival and growth rates

**Fig 3:**
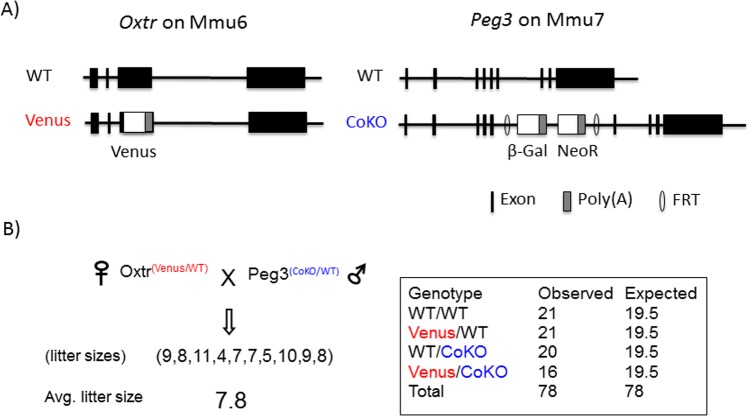
Schematic representations of mutant alleles and breeding schemes. (**A**) Targeted alleles of *Oxtr*^*venus*^ and *Peg3*^*CoKO*^. The 3rd exon of *Oxtr* has been replaced by an exogenous construct expressing the reporter Venus, thus the endogenous locus of *Oxtr* expresses the reporter Venus instead of the endogenous gene product, oxytocin receptor. In the case of *Peg3*^*CoKO*^, the 5th intron of *Peg3* has been inserted with an expression cassette containing β-Gal and NeoR along with Poly(A) signals, thus causing transcriptional truncation and subsequent loss of the PEG protein. (**B**) Breeding scheme. Female heterozygotes for *Oxtr*^*venus/+*^ were crossed with male heterozygotes for *Peg3*^*CoKO/+*^. The subsequent breeding results were presented with individual litter sizes, and also with a table summarizing the frequency of the four genotypes on right.

Potential involvement of *Peg3* in the expression and function of *Oxtr* was further tested through crossing two mutant mouse lines, *Oxtr*^*Venus/+*^ and *Peg3*^*CoKO/+*^ (**Fig 3A**). Both mutant alleles were generated using similar knock-in (KI) and knock-out (KO) strategies [11, 18]. The 3rd exon of *Oxtr* makes up the majority of the coding region (amino acid position 1–306 of the 388 amino-acid-long ORF), which has been replaced by an exogenous cassette expressing the reporter construct Venus. Thus, the mutant allele expresses the reporter protein instead of the endogenous gene product, oxytocin receptor. In the case of the *Peg3* locus, another cassette expressing β-Galactosidase (β-Gal) and Neomycin Resistance (NeoR) was inserted into the 5th intron of *Peg3*. In this mutant model, two sets of Poly(A) signals included in the expression cassette cause transcriptional truncation for the endogenous gene, thus resulting in the removal of PEG3 protein in the entire body [11, 12]. Since *Peg3* is expressed only from the paternal allele as an imprinted gene, paternal transmission of the mutant allele derives its phenotypic outcomes, such as reduced growth rates and defects in maternal-caring behaviors.

As a feasibility test, male heterozygotes for *Peg3*^*CoKO/+*^ were bred with female heterozygotes for *Oxtr*^*Venus/*+^, deriving 10 litters of 78 pups (**Fig 3B**). The average litter size, 7.8, appeared to be normal given the genetic background of the two mutant strains, C57BL/6J. The observed frequency of the four genotypes among the pups were also overall similar to those predicted based on mendelian inheritance pattern. Although the frequency of the pups with double heterozygote, *Oxtr*^*Venus/*+^; *Peg3*^*+/CoKO*^, was slightly smaller than the expected, 16 versus 19.5, there was no statistical significance. Thus, this suggests that the pups with the four genotypes are all viable without any major phenotypic consequence. According to previous studies, the paternal transmission of *Peg3*^*CoKO/+*^ has been shown to cause reduced body size among the mutant pups [9–13]. Consistent with this, we also observed reduced body weights among the two groups of the pups, the first group (*Oxtr*^+/+^; *Peg3*^*+/CoKO*^) and the second group (*Oxtr*^*Venus/*+^; *Peg3*^*+/CoKO*^). However, we have not observed any weight difference between these two genotypes, indicating that the mutation on *Oxtr* may not have any additional effect on the reduced growth rates of the animals that had already been impacted by the mutation on *Peg3*.

### Mutational effects of *Peg3* on the expression of *Oxtr* in the mammary gland

**Fig 4:**
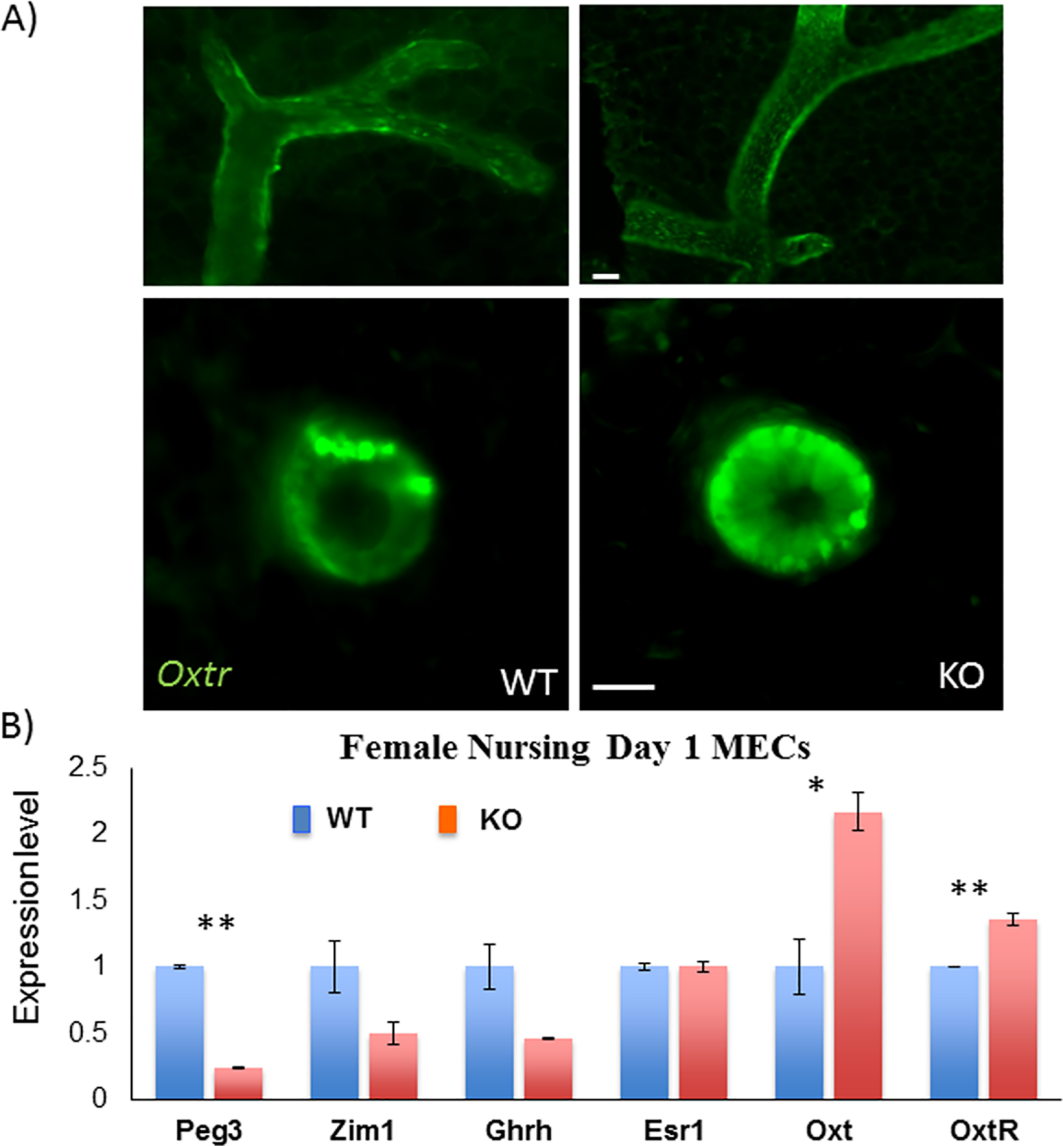
Up-regulation of *Oxtr* in the mammary gland of nursing females with *Peg3*^*+/CoKO*^. (**A**) At one day postpartum, a set of mammary glands were harvested from the following two females: *Oxtr*^*Venus/+*^; *Peg3*^*+/+*^ (WT) and *Oxtr*^*Venus/+*^; *Peg3*^*+/CoKO*^ (KO). The spatial expression patterns of *Oxtr* were monitored through the signal of the reporter Venus, and the intensity of signals were also compared between WT and KO. The top panels represent the excretory ducts, while the bottom panels show the secretory acini of the mammary gland of the WT and KO mice. Lining epithelial cells show higher levels of *Oxtr* immunofluorescence in KO than in WT. The scale bars for both panels are 20 μm. (**B**) qRT-PCR analyses. Total RNA was isolated from the mammary epithelial cells of female littermates with the following genotypes: *Oxtr*^*+/+*^; *Peg3*^*+/+*^ (WT) and *Oxtr*^*+/+*^; *Peg3*^*+/CoKO*^ (KO). These RNA were used for cDNA synthesis, which were subsequently used for qRT-PCR analyses. The expression levels of each gene were compared between WT and KO, and the observed differences were presented with statistical significance (*, *p* <0.01 and **, *p* < 0.001).

**Fig 5:**
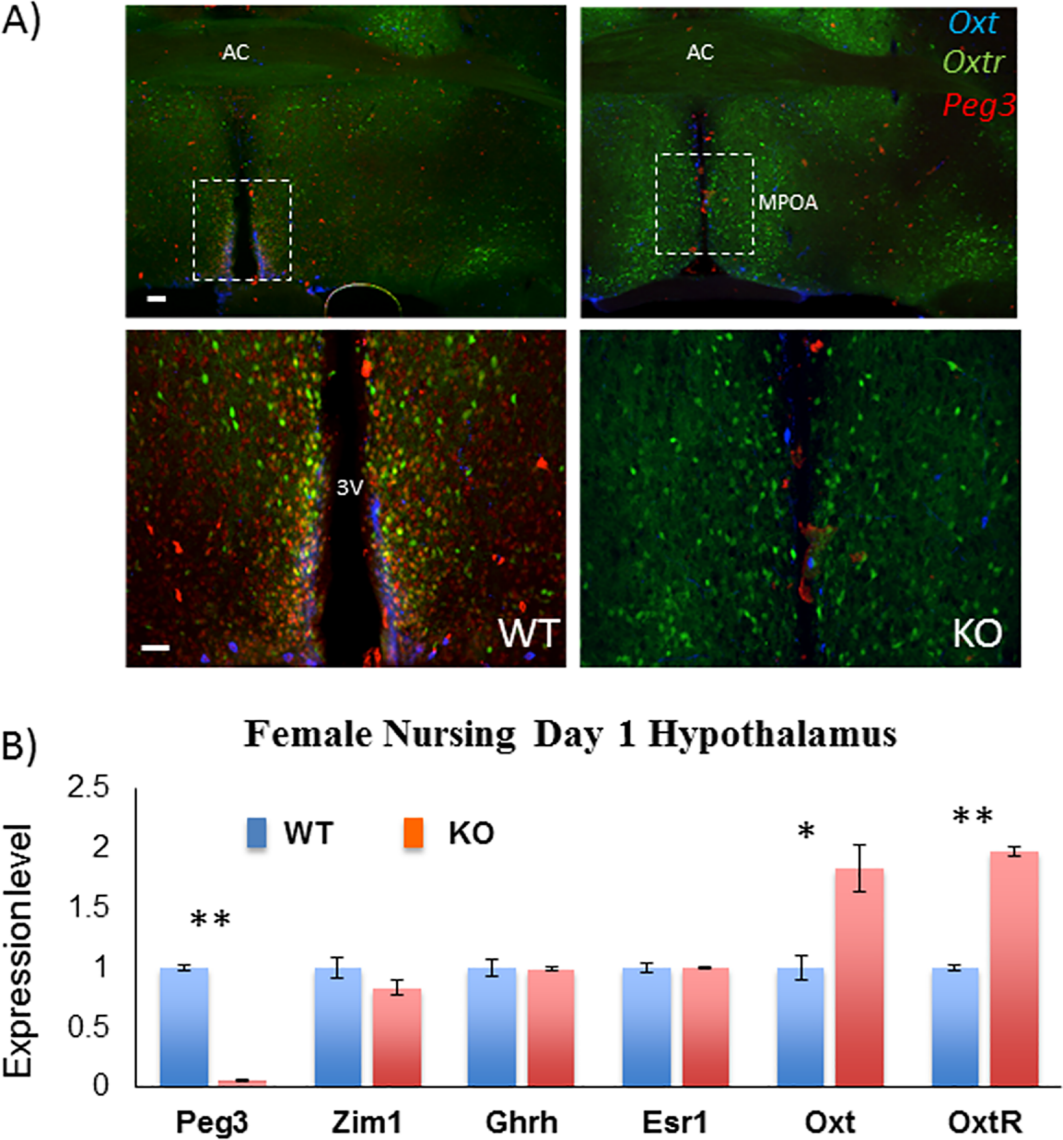
Up-regulation of *Oxtr* in the hypothalamus of nursing females with *Peg3*^*+/CoKO*^. (**A**) At one day postpartum, a set of brains were harvested from the following two females: *Oxtr*^*Venus/+*^; *Peg3*^*+/+*^ (WT) and *Oxtr*^*Venus/+*^; *Peg3*^*+/CoKO*^ (KO). The harvested brains were fixed, sectioned and immunostained with anti-OXT antibody (blue) and anti-PEG antibody (red). The expression of *Oxtr* was monitored through the signal of the reporter Venus (green). The top panels represent a low power view of the hypothalamic region, while the bottom panels show a high power view of the boxed regions on top panels. *Oxtr*-Venus^+^ cells were localized along the 3^rd^ ventricle and almost all co-localized with *Peg3* in WT mice. *Oxtr*-Venus expression in KO mice was much broader and extended into the medial preoptic area. The scale bars for top and bottom panels are 0.5 mm and 200 μm, respectively. AC: Anterior Commisure, 3V: 3^rd^ Ventricle, MPOA: Medial PreOptic Area. (**B**) qRT-PCR analyses. Total RNA was isolated from the hypothalamus of female littermates with the following genotypes: *Oxtr*^*+/+*^; *Peg3*^*+/+*^ (WT) and *Oxtr*^*+/+*^; *Peg3*^*+/CoKO*^ (KO). These RNA were used for cDNA synthesis, which were then used for qRT-PCR analyses. The expression levels of each gene were compared between WT and KO, and the observed differences were presented with statistical significance (*, *p* <0.01 and **, *p* < 0.001).

The females generated from the breeding experiments were also used to examine the mutational effects of *Peg3* on the expression levels and patterns of *Oxtr* (**Fig 4 and Fig 5**). For this series of analyses, we focused on the two major organs that are involved in milk letdown, the mammary gland and hypothalamus [14, 15]. The tissues were also harvested from the dams at one day postpartum, since this time-point should be critical for establishing the milk letdown process in the dams. We set up several sets of breeding pairs with each housing one wild-type male along with two female littermates with the following genotypes: *Oxtr*^*Venus/*+^; *Peg3*^*+/+*^ and *Oxtr*^*Venus/*+^; *Peg3*^*+/CoKO*^. These two types of females will be simply referred to as ‘WT’ and ‘KO’ hereafter based on the genotype of *Peg3*, respectively. At one day postpartum, the mammary glands were harvested from the two females, WT and KO, and the harvested tissues were fixed, and then used for inspecting the spatial expression pattern of *Oxtr* (**Fig 4A**). Overall, we observed a higher density of Venus-positive (Venus^+^) cells lining the mammary ducts as well as an increase in Venus^+^ cells along the basal region of cross-sectioned mammary ductal structures in KO than in WT samples (**Fig 4A**). To further follow up this observation, another set of the mammary glands were harvested from one-day-postpartum female littermates with the following genotypes: *Oxtr*^*+/*+^; *Peg3*^*+/+*^ (WT) and *Oxtr*^*+/*+^; *Peg3*^*+/CoKO*^ (KO). These mammary glands were used for further isolating mammary epithelial cells (MECs), which were used for isolating total RNA and subsequent cDNA synthesis. This set of cDNA was then used for another series of expression analyses involving qRT-PCR (**Fig 4B**). As expected, the expression levels of *Peg3* were significantly down-regulated in the KO sample compared to the WT sample. This was also the case for another imprinted gene *Zim1* (Zinc finger gene 1 imprinted) and *Ghrh* (Growth hormone-releasing hormone). In the case of *Esr1* (Estrogen receptor alpha), there was no difference between WT and KO. In contrast, the expression levels of both *Oxtr* and *Oxt* were up-regulated in the KO sample, 1.4-fold and 2.1-fold, respectively. The observed up-regulation of *Oxtr* appeared to be consistent with the greater density of Venus^+^ signals detected from the KO sample (**Fig 4A**). Thus, this series of analyses concluded that the expression levels of *Oxtr* were up-regulated in the mammary gland of the KO sample, particularly in the basal region of mammary epithelial cells.

### Mutational effects of *Peg3* on the expression of *Oxtr* in the hypothalamus

We also performed a similar series of expression analyses using the hypothalamus that had been harvested from the same sets of the females with the two genotypes, WT and KO (**Fig 5**). For this series of analyses, the harvested brains were first fixed, sectioned and finally immunostained with the following antibodies: anti-OXT and anti-PEG3 antibodies. The expression of *Oxtr* was monitored again through the signal of the reporter Venus. As expected, the expression of *Peg3* was not detectable at all throughout the entire area of the hypothalamus in the KO sample. On the other hand, the spatial expression patterns of *Oxtr* were quite different between WT and KO samples. In the WT sample, the expression of *Oxtr* was mainly detected from the neuronal cells that are localized immediately adjacent to the 3rd ventricle column. In contrast, the expression of *Oxtr* in the KO sample was detected from the neuronal cells that are localized in the much broader region of the hypothalamus, suggesting that the mutation on *Peg3* may result in an ectopic expression of *Oxtr* within the hypothalamus. A series of sections derived from the different areas of the hypothalamus were also inspected, showing similar ectopic expression of *Oxtr* (**S2 File**). In the case of *Oxt*, we have not detected any significant difference between WT and KO samples in terms of its spatial expression pattern (**S3 File**).

We also performed a series of qRT-PCR to measure the expression level differences of several genes between *Oxtr*^*+/*+^; *Peg3*^*+/+*^ (WT) and *Oxtr*^*+/*+^; *Peg3*^*+/CoKO*^ (KO) samples (**Fig 5B**). According to the results, the expression levels of *Peg3* were not detectable in KO, consistent with the results from previous studies [11, 12]. The expression levels of the three genes, including *Zim1*, *Ghrh*, and *Esr1*, showed no major difference between WT and KO samples. On the other hand, the expression levels of *Oxtr* and *Oxt* were similarly up-regulated in the KO sample compared to those of the WT sample. The observed up-regulation of *Oxtr* appeared to agree with the ectopic expression pattern detected through the immunostaining (**Fig 5A**). This series of analyses were repeated three times using three biological replicates. Thus, three independent trials with two different methods confirmed similar up-regulation of *Oxtr* in the KO sample. Overall, this series of analyses demonstrated that the mutation on *Peg3* results in the up-regulation of *Oxtr* in the hypothalamus, which is also similar to the outcome observed in the mammary gland.

## Discussion

In the current study, we characterized the *in vivo* binding of PEG3 to a genomic target that is located within the 3rd exon of the mouse *Oxtr* locus. Also, the expression analyses using a combination of two mutant mouse models, Venus and CoKO, further demonstrated that removal of PEG3 resulted in the up-regulation of *Oxtr* in mammary epithelial cells and in the hypothalamus. Thus, this suggests that *Peg3* may play roles as a transcriptional regulator for the expression of *Oxtr*.

The results presented here demonstrated that PEG3 may directly control the expression of *Oxtr*, as a DNA-binding protein (**Fig 1 and Fig 2**). This also agrees with the previous studies that PEG3 functions as a DNA-binding protein controlling the expression of several downstream genes, including *Pgm2l1*, *H19*, *Msl1* and *Msl3* [4–7]. PEG3 has been predicted to be a transcriptional repressor, since the known downstream genes tend to be up-regulated in the mutant samples lacking PEG3 [6, 7]. This turned out to be the case for *Oxtr*. In the mammary gland, the KO sample displayed 1.4-fold up-regulation of *Oxtr* based on the results from qRT-PCR, and also exhibited much more dense signals within the location of mammary epithelial cells (**Fig 4A**). In the hypothalamus, the overall expression levels of *Oxtr* were also 2-fold up-regulated in the KO sample. Interestingly, the immunostaining showed that the neuronal cells localized in the broader region adjacent to the 3rd ventricle ectopically expressed *Oxtr* in the KO sample. A similar observation was also observed from the previous study that several placenta-specific gene families are ectopically de-repressed in the hypothalamus of the mutant mice [11]. In that regard, it is also interesting to note that *Peg3* is known to be expressed more preferentially in the vasopressinergic neurons than in the oxytocinergic neurons in the hypothalamus of rats [18]. If this is the case, the observed up-regulation of *Oxt* might also be an outcome of the ectopic expression in the KO model, although we have not been able to confirm this possibility (**S3 File**). Thus, PEG3 may play a transcriptional repressor role not only for the cells that already express *Oxtr*, such as the epithelial cells in the mammary gland, but also for the cells that do not usually express *Oxtr*, as seen in the neuronal cells in the hypothalamus. Overall, the current study clearly demonstrated that PEG3 functions as a transcriptional repressor for *Oxtr*, and further suggests that this repressor function of *Peg3* may be cell type- and context-dependent.

Several mutant models targeting *Peg3* are known to have defects in the milk letdown process, and these defects have been, so far, believed to be associated with a loss of OXT protein levels [9–12]. Yet, the current study indicated that the mutant animals lacking PEG3 have increased levels of *Oxtr* and *Oxt* (**Fig 4 and Fig 5**). It is somewhat enigmatic how the up-regulated oxytocin circuitry might cause defects in the milk letdown process. Two scenarios are possible. First, the relative amount of OXTR and OXT to the other proteins may be optimized and tightly controlled as part of some signaling pathways. In this case, the excessive amount of OXTR could have a diluting effect on the downstream proteins that are involved in these signaling pathways, thus causing a defect in the milk letdown process. Second, it is also feasible to predict that the hormone OXT might be produced just sufficient for the cells participating in the oxytocin circuitry. In this situation, if many unrelated cells happen to also express *Oxtr* due to its de-repression, this might cause a similar diluting effect on the downstream proteins involved in the signaling pathways. This is feasible, since *Oxtr* was found to be ectopically expressed in the neuronal cells within the hypothalamus of the KO sample (**Fig 5A**). Overall, although very speculative at the moment, it should be very interesting to investigate these possibilities in the near future. On a separate note, it is relevant to note that the expression of *Oxt* and *Oxtr* in other tissues, such as the gastrointestinal tract, may also be associated with maternal care defects and gastrointestinal motility in rat models [19]. Although the current study has mainly focused on the hypothalamus and mammary gland, potential connection of the oxytocin circuitry to the enteric nervous system may also offer some insight into the defects observed in Peg3-deficient mice.

## Materials and methods

### Ethics statement

All the experiments related to mice were performed in accordance with National Institutes of Health guidelines for care and use of animals, and also approved by the Louisiana State University Institutional Animal Care and Use Committee (IACUC), protocol #16–060.

### Mouse breeding

In the current study, we used two mutant strains that have been previously characterized: the *Oxtr*^*Venus/+*^ and *Peg3*^*CoKO/+*^ strains [11, 20]. Female heterozygotes for *Oxtr*^*Venus/+*^ were crossed with male heterozygotes for *Peg3*^*CoKO/+*^. The subsequent pups were analyzed in terms of sex, genotype and weight. For genotyping, genomic DNA was isolated from either clipped ears or tail snips by incubating the tissues overnight at 55°C in the lysis buffer (0.1 M Tris-Cl, pH 8.8, 5 mM EDTA, pH 8.0, 0.2% SDS, 0.2 M NaCl, 20 μg/ml Proteinase K). The isolated DNA was subsequently genotyped using the following two sets of primers: for the *Oxtr*^*Venus/+*^ strain, Primer F (5’-GTTGGGAACAGCGGTGATTA-3’) and R (5'-GGCTCAGGCTTTCTCTACTT-3'); for the *Peg3*^*CoKO/+*^ strain, Peg3-5arm (5’-CCCTCAGCAGAGCTGTTTCCTGCC-3’) and LAR3 (5’-CAACGGGTTCTTCTGTTAGTCC-3’). The sex of the pups was determined through PCR using the following primer set: mSry-F (5’-GTCCCGTGGTGAGAGGCACAAG-3’) and mSry-R (5’-GCAGCTCTACTCCAGTCTTGCC-3’).

### Chromatin ImmunoPrecipitation (ChIP)

Chromatins were prepared from MEF and adult brains, according to the method previously described [6]. In brief, the homogenized samples were first cross-linked with 1% formaldehyde for 20 minutes, and then lysed with buffer containing protease inhibitor cocktail (Cat. No. 539131, Millipore). The subsequent nuclei were fractionated with sonication to derive a pool of DNA fragments size-ranging from 300 to 500 bp in length. The prepared chromatin was immunoprecipitated with a commercial anti-PEG3 antibody (Cat. No. ab99252, abcam). The immunoprecipitated DNA was dissolved in 100 μl of TE for PCR analyses.

### Electrophoretic Mobility Shift Assay (EMSA)

EMSA was performed using a gel shift assay system kit (Cat. No. E3053, Promega). This series of assays used mouse brain nuclear extract (Cat. No. 36053, Active Motif). For the competition assays, competitor oligonucleotide duplexes (1.74 pico mole, 200X) were first incubated with mouse brain nuclear extract (2.72 μg) at room temperature for 10 minutes. Later, the P^32^-labeled Pgm2l1 oligonucleotide duplex probe (8.7 fento mole, 1X) was added and incubated at room temperature for additional 20 minutes. The reaction mixture was separated on a 5% TBE gel (Cat. No. 456–5014, Bio-Rad), and exposed to a film for 2 to 6 hours at -80°C.

### Isolation of mammary epithelial cells

Female dams were sacrificed using CO_2_ asphyxiation at one day postpartum. Mammary glands were extracted without lymph nodes, and placed in a 50 mL conical tube with 10 mL of DMEM/F12 1:1 media. The harvested mammary glands were transferred to petri dishes and minced to a size of ~1 mm^3^ under a laminar flow hood. The minced tissue was transferred to a new 50 mL conical tube containing 10 mL of DMEM/F12 1:1 supplemented with Collagenase (300 U/mL) and Hyaluronidase (100 U/mL), and placed on a 37°C shaking incubator at 200 RPM for 2 hours. The mixture was spun down at 2000 RPM for 5 minutes, and the subsequent pellet was recovered from the supernatant containing the lipid layer. The pellet was again resuspended in 10 mL of HBSS. Red blood and stromal cells were depleted with 3 rounds of pulse centrifugations at 1500 RPM for 7 seconds. After the three rounds of wash, the final pellet was resuspended in 10 mL of HBSS, partitioned into two 5 mL aliquots, and spun down again at 2000 RPM for 5 minutes. These two fractions were used for RNA isolation and subsequent cDNA synthesis.

### Quantitative qRT-PCR analyses

Total RNA was isolated from the mammary epithelial cells and the hypothalamus of adult mice using a commercial kit (Trizol, Invitrogen). The total RNA was reverse-transcribed using the M-MuLV kit (Invitrogen), and the subsequent cDNA was used as a template for quantitative real-time PCR. This analysis was performed with the iQ SYBR green supermix (Bio-Rad) using the ViiA*™* 7 Real-Time PCR System (Life Technologies). All qRT-PCR reactions were carried out for 40 cycles under standard PCR conditions. The analyses of the results from qRT-PCR were described previously [21]. Statistical significance of potential difference of expression levels of a given gene between two samples was tested with Mann-Whitney U test. The information regarding individual primer sequences is available through previous studies [11].

### Immunocytochemistry

At one day postpartum, three sets of nursing females with the following genotypes, *Oxtr*^*Venus/*+^; *Peg3*^*+/+*^ (WT) and *Oxtr*^Venus/+^; *Peg3*^+/CoKO^ (KO), were anesthetized with Ketamine-Xylazine (9:1; 100 mg/kg; i.p.) and perfused through the heart with 0.01 M sodium phosphate buffered saline (PBS; pH 7.2), followed by 4% paraformaldehyde in 0.1 M sodium phosphate buffer (PB; pH 7.2). The brains were extracted and post fixed in the same fixative for overnight. Coronal sections were transected at 40 μm by an automated vibratome (Leica VT1200; Mannheim, Germany).

To enhance the signal of Venus, immunocytochemical localization of Venus with anti-green fluorescent protein (GFP) antibody (ab13970; abcam, Cambridge, UK) was conducted in the brain sections. The free-floating brain slices were incubated with the primary antibodies against GFP at dilutions of 1:10,000 in PBS containing 0.5% Triton X-100 (PBST) with continuous gentle agitation at 4°C for overnight. The brain sections were subsequently incubated with a secondary antibody (goat anti-chicken) conjugated with Alexa Fluor 488 (Jackson ImmunoResearch, West Grove, PA) at room temperature for 4 hours. The sections were also incubated with anti-PEG3 at dilutions of 1:10,000 (custom-made antibody, [6]) and monoclonal anti-OT-neurophysin antibody (PS38 provided by H. Gainer, NIH) at 1:500 overnight at 4°C, and labeled with AffiniPure Goat Anti-Rabbit IgG Alexa Fluor647 and AfiiniPure Goat Anti-Mouse IgG DyLight549 respectively (Jackson ImmunoResearch, West Grove, PA) at 1:250 dilution at room temperature for 2–4 hours. The sections were mounted in polyvinyl alcohol (PVA) with anti-fading agent 1,4-Diazabicyclo[2.2.2]octane (DABCO) that consists of 4.8 g PVA, 12 g glycerol, 12 ml dH_2_O, 24 ml 0.2 M Tris-HCl, and 1.25 g DABCO. Fluorescence microscopic images (1280 x 1024) were acquired digitally (Eclipse 80i equipped with DS-QiMc, Nikon, Tokyo, Japan).

## Supporting information

S1 File PEG3's ChIP-seq profile in the 20-kb genomic region surrounding the *Oxt* and *Avp* loci. This file contains the image showing the Peg3 ChIP-seq results as bigwig files as tracks below the 20-kb genomic region harboring the *Oxt* and *Avp* genes.
(PPTX)

S2 File *Oxtr* expression within the hypothalamus between WT and KO. This file contains a set of images showing the ectopic expression of *Oxtr* in the medial preoptic area of the hypothalamus, which is separate from those presented in **Fig 5**.
(PPTX)

S3 File *Oxt* expression in the SON and PVN of WT and KO. This file contains a set of images comparing the expression of *Oxtr* and *Oxt* within the PVN and SON areas between the females with the following genotypes: *Oxtr*^*Venus/+*^; *Peg3*^*+/+*^ (WT) and *Oxtr*^*Venus/+*^; *Peg3*^*+/CoKO*^ (KO).
(PPTX)
