# Supplementary figures and images for: Oxytocin receptor is regulated by Peg3
Source: PLoS One. 2018 Aug 14;13(8):e0202476. doi: 10.1371/journal.pone.0202476 (PMC6091971; doi:10.1371/journal.pone.0202476)

## Slide 1
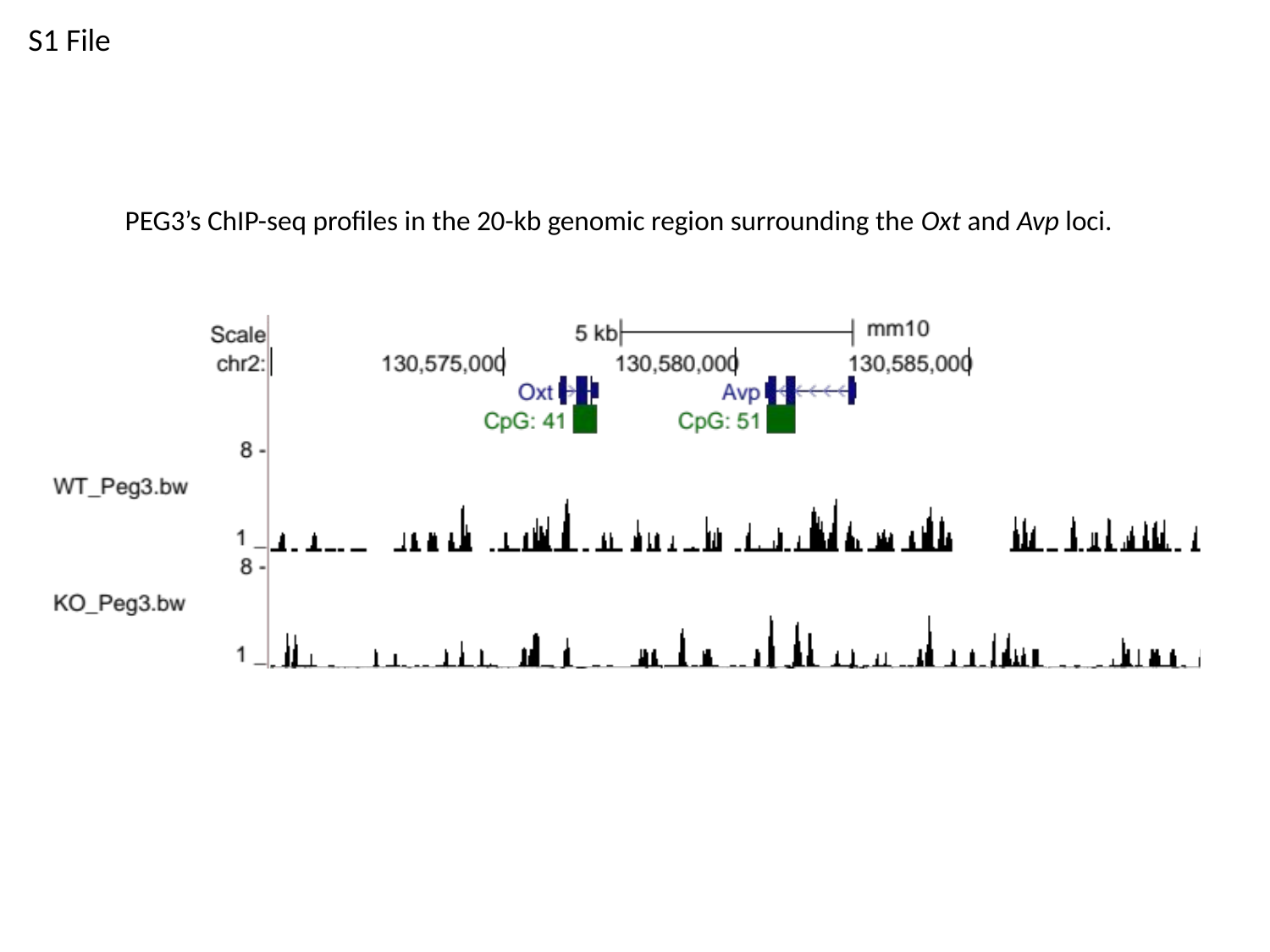

S1 File
PEG3’s ChIP-seq profiles in the 20-kb genomic region surrounding the Oxt and Avp loci.

Supplement: S1 File — This file contains the image showing the Peg3 ChIP-seq results as bigwig files as tracks below the 20-kb genomic region harboring the Oxt and Avp genes. (PPTX) [file pone.0202476.s001.pptx]
