# Supplementary material for: Oxytocin receptor is regulated by Peg3
Source: PLoS One. 2018 Aug 14;13(8):e0202476. doi: 10.1371/journal.pone.0202476 (PMC6091971; doi:10.1371/journal.pone.0202476)
Supplement: S2 File — This file contains a set of images showing the ectopic expression of Oxtr in the medial preoptic area of the hypothalamus, which is separate from those presented in Fig 5. (PPTX) [file pone.0202476.s002.pptx]

## Slide 1
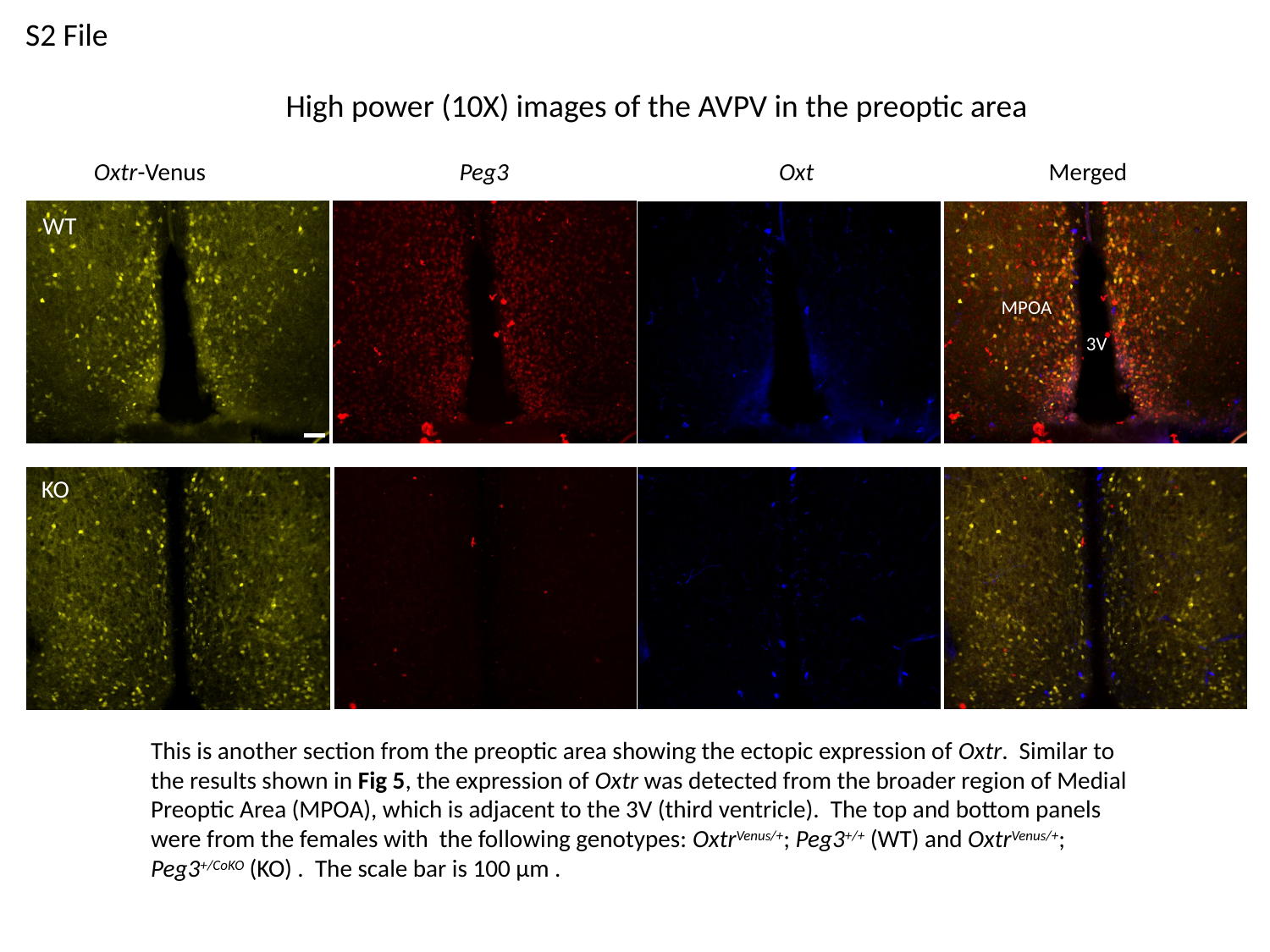

S2 File
High power (10X) images of the AVPV in the preoptic area
Oxtr-Venus
Peg3
Oxt
Merged
WT
MPOA
3V
KO
This is another section from the preoptic area showing the ectopic expression of Oxtr. Similar to the results shown in Fig 5, the expression of Oxtr was detected from the broader region of Medial Preoptic Area (MPOA), which is adjacent to the 3V (third ventricle). The top and bottom panels were from the females with the following genotypes: OxtrVenus/+; Peg3+/+ (WT) and OxtrVenus/+; Peg3+/CoKO (KO) . The scale bar is 100 μm .
