# Supplementary material for: Oxytocin receptor is regulated by Peg3
Source: PLoS One. 2018 Aug 14;13(8):e0202476. doi: 10.1371/journal.pone.0202476 (PMC6091971; doi:10.1371/journal.pone.0202476)
Supplement: S3 File — This file contains a set of images comparing the expression of Oxtr and Oxt within the PVN and SON areas between the females with the following genotypes: OxtrVenus/+; Peg3+/+ (WT) and OxtrVenus/+; Peg3+/CoKO (KO). (PPTX) [file pone.0202476.s003.pptx]

## Slide 1
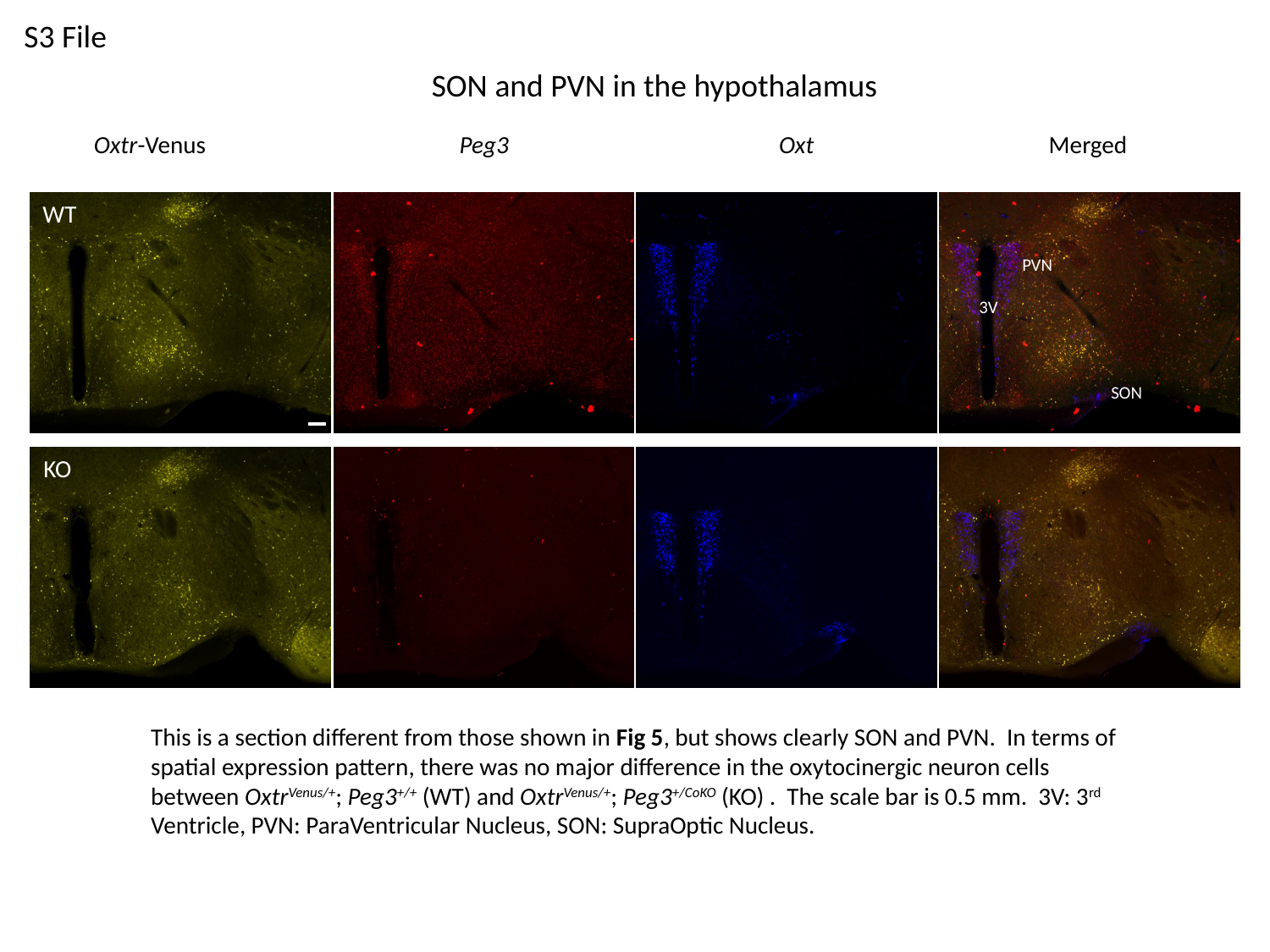

S3 File
SON and PVN in the hypothalamus
Oxtr-Venus
Peg3
Oxt
Merged
WT
PVN
3V
SON
KO
This is a section different from those shown in Fig 5, but shows clearly SON and PVN. In terms of spatial expression pattern, there was no major difference in the oxytocinergic neuron cells between OxtrVenus/+; Peg3+/+ (WT) and OxtrVenus/+; Peg3+/CoKO (KO) . The scale bar is 0.5 mm. 3V: 3rd Ventricle, PVN: ParaVentricular Nucleus, SON: SupraOptic Nucleus.
